# The influence of caregiver attitudes and socioeconomic group on formal and informal mental health service use among youth

**DOI:** 10.1192/j.eurpsy.2022.24

**Published:** 2022-06-10

**Authors:** Cristiane Silvestre Paula, Carolina Ziebold, Wagner S. Ribeiro, Pedro Mario Pan, Jair Jesus Mari, Rodrigo Bressan, Euripedes Constantino Miguel, Luiz Augusto Rohde, Giovanni A. Salum, Sara Evans-Lacko

**Affiliations:** 1 Programa de Pós-Graduação em Distúrbios do Desenvolvimento e Centro Mackenzie de Pesquisa sobre a Infância e Adolescência, Universidade Presbiteriana Mackenzie (UPM), Rua da Consolação 930, Edifício 28, São Paulo, Brazil; 2 Departamento de Psiquiatria, Universidade Federal de São Paulo (UNIFESP), São Paulo, Brazil; 3 Care Policy and Evaluation Centre, London School of Economics and Political Science (LSE), London, United Kingdom; 4 Laboratório de Neurociências Integrativas (LiNC), Departamento de Psiquiatria, Universidade Federal de São Paulo (UNIFESP), São Paulo, Brazil; 5 Instituto Ame Sua Mente, São Paulo, Brazil; 6 Departamento de Psiquiatria, Faculdade de Medicina da Universidade de São Paulo (FMUSP), São Paulo, Brazil; 7 ADHD Outpatient Program & Developmental Psychiatry Program, Hospital de Clínicas de Porto Alegre, Federal University of Rio Grande do Sul, Porto Alegre, Brazil; 8 National Institute of Developmental Psychiatry, São Paulo, Brazil; 9 Section on Negative Affect and Social Processes, Hospital de Clínicas de Porto Alegre, Universidade Federal do Rio Grande do Sul, Porto Alegre, Brazil

**Keywords:** Child Mental Health, Service Use, Formal Care, Informal Care, Stigma, Brazil

## Abstract

**Background:**

Young people can receive mental health care from many sources, from formal and informal sectors. Caregiver characteristics/experiences/beliefs may influence whether young people get help and the type of care or support used by their child. We investigate facilitators/barriers to receiving formal and/or informal care, particularly those related to the caregiver’s profile.

**Methods:**

We interviewed 1,400 Brazilian primary caregivers of young people (aged 10–19), participants of a high-risk cohort. Caregivers reported on young people’s formal/informal mental health care utilization, and associated barriers and facilitators to care. Data were also collected on youth mental health and its impact on everyday life; and caregiver characteristics—education, socioeconomics, ethnicity, mental health, and stigma. Logistic regression models were used to examine the relationship between caregiver and young people characteristics with formal/informal care utilization.

**Results:**

Persistence and greater impact of youth mental health conditions were associated with a higher likelihood of care, more clearly for formal care. Caregiver characteristics, however, also played a key role in whether young people received any care: lower parental stigma was associated with greater formal service use, and lower socioeconomic class showed higher odds of informal care (mainly from religious leaders).

**Conclusions:**

This study highlights the key role of the caregivers as gatekeepers to child treatment access, particularly parental stigma influencing whether young people received any mental health care, even in a low resource setting. These results help to map barriers for treatment access and delivery for young people, aiming to improve intervention efforts and mental health support.

## Introduction

Mental health problems in young people are prevalent, and have a significant impact on their lives [1], but often they do not receive appropriate care [2]. Support and treatment for such problems can arise from formal and informal sources of care such as religious leaders, non-health professionals, and self-help [3]. Support often involves multiple, ideally coordinated, sources of care from health, education, and third-sector organizations [4]. Most studies investigating access to mental health care among youth, however, use clinical samples and focus on barriers to any service contacts rather than considering the context, type, and appropriateness or how the care meets the needs of individuals. Moreover, the range of sources of informal care makes systematic tracking of utilization difficult, and the types of support provided may not be recognized as care [5].

The receipt of adequate mental health by young people depends on a range of factors. *First*, youth are rarely able to seek and access care on their own; therefore, caregivers often represent key gatekeepers to care. Caregiver characteristics (e.g., social class and their own psychopathology) and experiences related to mental health (e.g., their knowledge, beliefs, and potential prejudice/stigma toward mental health) can influence their children’s pathway to care [6, 7]. *Second*, characteristics of the youth (e.g., gender, age, and psychopathology) can influence caregiver perceptions of their children’s need for care, consequently influencing help-seeking [6]. In addition to caregiver and youth characteristics, structural barriers, such as cost, distance, and limitations of the appointment system, are important factors in the care pathway [8, 9].

Therefore, characterizing formal and informal sources of support and understanding factors linked to care access and ongoing treatment can help policymakers to map barriers and facilitators for treatment access and delivery to have a more detailed picture of the needs of a population aiming at improving intervention efforts maximizing mental health provision.

This study aims: (a) to describe types of formal and informal mental health-related services used by youth in Brazil; (b) to investigate facilitators and barriers to any versus no contact with formal and/or informal care, particularly those related to caregivers’ and young people characteristics; and (c) given that barriers to any contact may differ from the barriers among those receiving some type of support, we also investigated perceived barriers to care among those who had at least some service contact.

We hypothesized that low socioeconomic group (SEG), the presence of caregiver mental health problems, and caregiver stigma would be associated with a lower likelihood of formal or informal child mental health service use.

## Method

### Sample

This study was nested within the Brazilian High-Risk Cohort Study for Mental Conditions (BHRCS) [10], which is a large school-based community cohort enriched for mental health conditions. The aim of the study was to understand the developmental trajectories of psychopathology and mental disorders using a two-stage design. Families were recruited from 22 schools in Porto Alegre and 35 schools in São Paulo. First, 9,937 parents were screened using the Family History Survey (FHS) on the registry day. The FHS is used to screen all family members (in 87% of cases, the mother was the primary informant) for the Diagnostic and Statistical Manual of Mental Disorders, Fourth Edition (DSM-IV). An index of family load was computed for each of the potential eligible children based on the percentage of members in the family that screened positively for each of the disorders assessed, adjusted for relatedness. From this pool, we selected two subgroups: one randomly selected (*n* = 958) and one high-risk sample (*n* = 1,553), a subsample of children at increased risk of mental disorders based on FHS [10]. Because we would expect mental health service use to be relatively rare, this enriched sample, which included a high proportion of youth at-risk of mental health problems, is ideal. Moreover, as we wanted to understand the barriers among youth with mental health conditions who were not receiving care, we needed a nonclinical sample.

At baseline (2010–2011), 2,511 young people aged 6–14 years and their caregivers were interviewed. During the first follow-up (2014–2015), 2,010 parents/main guardians responded to a comprehensive assessment of mental health and mental health conditions related to their children (80% retention rate). However, for this study about use of services, we were only able to contact 94% of those who participated at the first follow-up (*n* = 1,881). Of those, 1,400 (74.4%) completed a further specific interview on mental health-related service use by their child (aged 10–19) and potential barriers and facilitators to using these services, 982 (70.1%) by telephone and 418 (29.9%) face-to-face. Of those, 1,400 (74.4%) completed a further specific interview on mental health-related service use by their child (aged 10–19) and potential barriers and facilitators to using these services, 982 (70.1%) by telephone and 418 (29.9%) face-to-face. Supplementary Figure S1 shows the sampling process flowchart. We compared characteristics of all baseline Brazilian High-Risk Cohort (BHRC) participants and those who completed the health-related service use study (Supplementary Table S1). Briefly, lower maternal education, lower socioeconomic status, Asian/indigenous ethnicity, female gender, and higher age at baseline increased the likelihood of attrition at follow-up.

All caregivers provided written consent, and all youth provided verbal assent. This study was approved by the Ethics committee of the UNIFESP (CAAE 06457219.9.0000.5505) and HC-Porto Alegre (CAAE 06457219.9.3001.5327).

### Measures

*Youth mental health service use:* The Service Assessment for Children and Adolescents-SACA [11, 12] was used to ask caregivers about service contacts in the previous year due to concerns regarding their children’s emotions and behavior problems, including alcohol- and drug-related problems. The SACA assesses types of mental health services used, including treatments received, reasons for service use, and its quality, including barriers and facilitators for accessing the services. It covers 30 service settings grouped in three areas: inpatient, outpatient, and school.

Overall, concordance between parental reports and records, and the test–retest reliability for 12-month service use of the SACA is moderate to substantial [12]. We received permission from the SACA developers to translate/adapt it for use in the Brazilian context in consultation with experts in the mental health system to ensure that we covered the relevant service types and settings.

For the current study, we first considered any formal or informal service use in the past 12 months and then subgroups of formal (health or education) and informal (religious/spiritual leader, self-help, and alternative therapies) mental health care (Supplementary Table S2).

*Barriers to sufficient mental health care among youth who received services:* As part of the SACA, caregivers who reported at least some service contact due to mental health problems in the past 12 months were asked: “Did you think the child needed any service other than the one she/he used?” Those who answered yes were asked about the reasons why the child had not received these services. The reasons included a list of 14 potential barriers and an open-ended question (“others”). Barriers were classified as: (a) structural (three items); (b) recognition and literacy (four items); (c) lack of trust and negative experiences with services/treatment (one item); and (d) stigma (one item; Supplementary Table S2).

*Trajectory of psychiatric diagnosis:* Psychiatric diagnoses were assessed at baseline and follow-up using the Brazilian-Portuguese version of the Development and Well-Being Assessment [13, 14], a structured interview used to generate DSM-IV diagnoses conducted by trained psychologists. At baseline, diagnostic assessment and interviews were performed with the caregiver only. At follow-up, diagnostic assessment considered caregiver reports and additional information from interviews with the youth about internalizing conditions. All responses were evaluated by trained psychiatrists who determined final diagnostic status (for more details, please see [10]). Three categories of diagnostic trajectories were created: (a) no diagnosis (no diagnosis at baseline neither at follow-up); (b) transient mental health condition (presence of diagnosis at baseline or follow-up); and (c) persistent mental health condition (presence of diagnosis at both time points).

*Impact of the mental health condition* was measured using the “impact supplement” of the Strengths and Difficulties Questionnaire (SDQ), which assesses the impact of behavioral and emotional difficulties on the young person’s life according to parental reports. This supplement begins with a general question about difficulties. For those with no difficulties, a score of 0 is attributed. For those with difficulties, the areas in which the difficulties occur are then explored. A total score is generated by summing five items: one item about distress, and four on social impairment in: (a) family life, (b) friendships, (c) learning, and (d) leisure activities. Higher scores represent greater impact. The impact score has demonstrated internal consistency, cross-informant correlations, and stability across time [15]. Moreover, other research suggests that the impact supplement adds information above and beyond symptoms [16].

*Caregiver education* (“Primary” [less than secondary education], “Secondary” [any secondary education], and “Higher” [any complete/incomplete university education]).

*SEG* was evaluated using the Brazilian Association of Research Companies questionnaire that classifies familial socioeconomic status according to purchasing power and head of household education [17], one of the most widely used questionnaires in Brazil, classifying families into eight SEGs, which were dichotomised into two categories: Middle-High and Middle-Low/Low.

*Caregiver ethnicity* was categorized into white, black, mixed (between white and black), and other (including Asian and Indigenous). Due to low numbers of Asian (*n* = 3) and Indigenous (*n* = 2) participants, these two categories were collapsed.

*Caregiver mental health* was assessed using the Kessler distress scale (K6) [18, 19], which is a self-administered scale assessing how frequently an adult experienced symptoms of psychological distress (e.g., how often did you feel hopeless?) in the past 30 days. It comprises six items and uses a five-point Likert scale, ranging from one (never) to five (all the time), where the higher the score, the greater the stress perceived by individual. The K6 has excellent internal consistency and reliability (Cronbach’s alpha = 0.89) [19] and it was used in the current study to screen non-specific serious mental illnesses among caregivers. Based on previous studies, caregivers who scored above 13 points on the K6 in the past 12-months were considered to have a mental health condition [20].

*Caregiver/parental stigma* was assessed using the intended behavior subscale of the Brazilian version of the Reported and Intended Behavior Scale (RIBS-BP). The RIBS-BP assesses future intended stigmatizing behavior across four contexts: living with, working with, living nearby, and continuing a relationship with someone with a mental health condition [21, 22]. Items are summed into a total score where higher scores reflect less stigma. The RIBS-BP showed good to excellent construct validity [22].

### Data analysis

First, we calculated the prevalence of each type of service utilization (any, formal, and informal) among youth, overall, and by trajectories of mental health condition and according to sociodemographic characteristics.

Then, we used logistic regression models to assess the association between caregiver characteristics (education, SEG, ethnicity, mental health, and parental stigma) and youth characteristics (gender, age, trajectory, and impact of the mental health condition) with utilization of formal and informal care. Unadjusted and adjusted odds ratios and 95% confidence intervals are presented.

Among those who received sufficient/insufficient mental health care, as defined above, we describe the additional types of barriers (structural, lack of recognition and illiteracy, lack of trust and negative experiences in services/treatment, anticipated stigma, and each of their subcategories), reported by the sample overall and according to trajectories of mental health condition (i.e., none, transient, and persistent). We then used logistic regression models to examine the association between factors related with reporting any additional barriers to sufficient mental health care. Due to the small sample size, we limited the number of variables included in this model to key caregiver (education, SEG, and mental health condition) and young person (gender, age, trajectory, and impact of mental health condition) characteristics. Exploratory analyses according to the type of barrier are presented in Supplementary Table S5. Data were analyzed using STATA/SE 16.0, version 16.

## Results

Participant characteristics are described in Supplementary Table S3. Table 1 presents the types of services used overall and by the trajectory of mental health condition. Across all mental health trajectory groups, formal service utilization was 6–7 times more common than informal care during the previous year. Most youth using informal care did so in combination with formal care, particularly among those with a persistent psychiatric diagnosis (83.3%).

**Table 1.** Mental health-related service use in the past 12 months among Brazilian young people, overall, and by the trajectory of mental health condition (*n* = 1,400).

	Full sample	No mental health condition	Transient mental health condition	Persistent mental health condition
	(*n* = 1,400)	(*n* = 861)	(*n* = 390)	(*n* = 149)
	*n* (%; 95% CI)	*n* (%; 95% CI)	*n* (%; 95% CI)	*n* (%; 95% CI)
Formal + informal				
*Any type of service utilization*	155 (11.1; 9.5–12.8)	45 (5.2; 3.9–6.9)	66 (16.9; 13.5–21.0)	44 (29.5; 22.8–37.3)
Formal				
*Health*				
Inpatient	8 (0.6; 0.3–1.1)	0 (0.0; −)	5 (1.3; 0.5–3.0)	3 (2.0; 0.7–6.1)
Emergency	4 (0.3; 0.1–0.8)	0 (0.0; −)	2 (0.5; 0.1–2.0)	2 (1.3; 0.3–5.2)
Specialty/specialist outpatient mental health care	117 (8.4; 7.0–9.9)	35 (4.1; 2.9–5.6)	45 (11.5; 8.7–15.1)	37 (24.8; 18.6–32.4)
General outpatient care	8 (0.6; 0.3–1.1)	2 (0.2; 0.1–0.9)	3 (0.8; 0.3–2.4)	3 (2.0; 0.7–6.1)
*Any type of health care utilization*	126 (9.0; 7.6–10.6)	36 (4.2; 3.0–5.7)	52 (13.3; 10.3–17.1)	38 (25.5; 19.1–33.1)
*Education*				
Therapy^a^	18 (1.3; 0.8–2.0)	4 (0.5; 0.2–1.2)	9 (2.3; 1.2–4.4)	5 (3.4; 1.4–7.8)
School assistance	12 (0.9; 0.5–1.5)	3 (0.4; 0.1–1.1)	4 (1.0; 0.4–2.7)	5 (3.4; 1.4–7.8)
Special classroom	5 (0.4; 0.2–0.9)	0 (0.0; −)	3 (0.8; 0.3–2.4)	2 (1.3; 0.3–5.2)
Special school	7 (0.5; 0.2–1.1)	1 (0.1; 0.0–0.8)	4 (1.0; 0.4–2.7)	2 (1.3; 0.3–5.2)
*Any type of school*	40 (2.9; 2.1–3.9)	8 (0.9; 0.5–1.9)	19 (4.9; 3.1–7.5)	13 (8.7; 5.1–14.5)
*Any type of formal care utilization*	145 (10.4; 8.9–12.1)	42 (4.9; 3.6–6.5)	60 (15.4; 12.1–19.3)	43 (28.9; 22.2–36.6)
Informal				
Religious	19 (1.4; 0.9–2.1)	5 (0.6; 0.2–1.4)	9 (2.3; 1.2–4.4)	5 (3.4; 1.4–7.8)
Self-help	4 (0.3; 0.1–0.8)	0 (0.0; −)	3 (0.8; 0.3–2.5)	1 (0.7; 0.1–4.6)
Alternative^a^	2 (0.2; 0.1–0.6)	1 (0.1; 0.0–0.8)	0 (0.0; −)	1 (0.7; 0.1–4.6)
*Any type of informal care utilization*	24 (1.7; 1.2–2.6)	6 (0.7; 0.3–1.5)	12 (3.1; 1.8–5.3)	6 (4.0; 1.8–8.7)

a One missing data.

Among formal service users, health services were used 3–4 times more often than educational services, across all mental health trajectory groups. The most frequent type of informal care was religious support (79.2%; Table 1).

After adjusting for youth mental health characteristics and caregiver characteristics, we found that parental stigma was the only caregiver characteristic associated with formal service use. Lower parental stigma was associated with higher odds of formal service use (i.e., each point on the RIBS-BP scale, increased the odds of formal service use by 12% [OR 95% CI: 1.04–1.91; *p*-value < 0.01]). Among young person characteristics, persistence of mental health conditions (OR: 3.15; 95% CI: 1.77–5.60; *p*-value < 0.01) and greater impact of mental health conditions on everyday life were associated with a greater likelihood of formal service use (i.e., each point on the SDQ impact scale increased odds of formal service use by 30% [OR 95% CI: 1.17–1.44; *p* < 0.01]; Table 2).

**Table 2.** Caregiver and child/adolescent characteristics associated with formal service use due to the trajectory of mental health condition in the past 12 months (bivariate and multivariable analyses; *n* = 1,400).

	Any formal service	No formal service	Unadjusted analysis		Adjusted multivariable analysis^a^	
	*n* (%; 95% CI)	*n* (%; 95% CI)	OR (95% CI)	*p*-value	OR (95% CI)	*p*-value
*Caregiver characteristics*						
Education						
Primary education (reference)	52 (8.4; 6.5–10.9)	568 (91.6; 89.2–93.6)				
Secondary education	71 (11.3; 9.1–14.1)	555 (88.7; 85.9–90.9)	1.40 (0.96–2.04)	0.08	1.36 (0.90–2.05)	0.14
Higher education	20 (13.6; 8.9–20.2)	127 (86.4; 79.8–91.1)	1.72 (0.99–2.98)	0.05	1.56 (0.83–2.96)	0.17
Social class						
Middle-high (reference)	54 (9.8; 7.5–12.5)	500 (90.3; 87.5–92.5)				
Middle-low/low	91 (10.8; 8.8–13.1)	755 (89.3; 87.0–91.2)	1.12 (0.78–1.59)	0.55	1.32 (0.87–2.00)	0.19
Ethnicity						
White (reference)	87 (11.1; 9.0–13.4)	703 (88.9; 86.6–91.1)				
Black	23 (11.9; 8.1–17.3)	163 (88.1; 82.7–92.0)	1.09 (0.67–1.78)	0.72	1.08 (0.63–1.85)	0.78
Mixed	35 (8.6; 6.2–11.7)	361 (91.4; 88.3–93.8)	0.76 (0.50–1.41)	0.18	0.91 (0.58–1.45)	0.70
Asian/indigenous	0 (0.0; −)	5 (100.0; −)	0.00 (–)	1.00	0.00 (–)	1.00
Mental health problems						
No (reference)	113 (9.4; 7.9–11.2)	1.085 (90.6; 88.8–92.1)				
Yes	32 (15.8; 11.4–21.6)	170 (84.2; 78.5–88.6)	1.81 (1.18–2.76)	<0.01	1.22 (0.76–1.97)	0.41
*Stigma M (SD; 95% CI)*	17.85 (2.8; 17.4–18.3)	17.14 (2.4; 17.0–17.3)	1.08 (1.01–1.14)	0.02	1.12 (1.04–1.91)	**<0.01**
*Young person characteristics*						
Gender						
Male (reference)	88 (11.0; 9.00–13.35)	713 (89.0; 86.7–91.0)				
Female	57 (9.5; 7.41–12.14)	542 (90.5; 87.9–92.6)	0.85 (0.60–1.21)	0.37	0.93 (0.63–1.36)	0.71
*Age M (SD; 95% CI)*	14.37 (2.0; 14.0–14.7)	14.53 (1.9; 14.4–14.6)	0.96 (0.88–1.05)	0.37	0.98 (0.89–1.08)	0.71
Mental health condition						
None (reference)	42 (4.9; 3.62–6.54)	819 (95.1; 93.5–96.4)				
Transient	60 (15.4; 12.13–19.32)	330 (84.6; 80.7–87.9)	3.55 (2.34–5.37)	<0.01	2.32 (1.48–3.63)	**<0.01**
Persistent	43 (28.9; 22.15–36.64)	106 (71.1; 63.4–77.9)	7.91 (4.94–12.67)	<0.01	3.15 (1.77–5.60)	**<0.01**
*Impact of mental health condition—M (SD; 95% CI)*	2.05 (2.3; 1.7–2.4)	0.61 (1.34; 0.5–0.7)	1.49 (1.40–1.63)	<0.01	1.30 (1.17–1.44)	**<0.01**

a Adjusted model with 1,385 participants; town of residence and data collection method (face-to-face or phone interview) were controlled.

SEG was the only caregiver variable associated with informal care: families from lower SEG (low-middle or low) had higher odds of informal care compared to families from higher SEG (OR: 4.51; 95% CI: 1.28–15.91; *p*-value = 0.02; Table 3).

**Table 3.** Caregiver and child/adolescent characteristics associated with informal service use due to the trajectory of mental health condition in the past 12 months (bivariate and multivariable analyses; *n* = 1,400).

	Any informal service	No informal service	Unadjusted analysis		Adjusted multivariable analysis^a^	
	*n* (%; 95% CI)	*n* (%; 95% CI)	OR (95% CI)	*p*-value	OR (95% CI)	*p*-value
*Caregiver characteristics*						
Education						
Primary education (reference)	10 (1.6; 0.9–3.0)	610 (98.4; 97.0–99.1)				
Secondary education	13 (2.1; 1.2–3.5)	613 (97.9; 96.5–98.8)	1.29 (0.56–2.97)	0.54	1.47 (0.61–3.51)	0.39
Higher education	1 (0.7; 0.1–4.7)	146 (99.3; 95.3–99.9)	0.42 (0.53–3.29)	0.41	0.64 (0.07–5.61)	0.69
Social class						
Middle-high (reference)	3 (0.5; 0.2–1.7)	551 (99.5; 98.3–99.8)				
Middle-low/low	21 (2.5; 1.6–3.8)	825 (97.5; 96.2–98.4)	4.68 (1.39–15.75)	0.01	4.51 (1.28–15.91)	**0.02**
Ethnicity						
White (reference)	14 (1.8; 1.1–3.0)	776 (98.2; 97.0–99.0)				
Black	2 (1.0; 0.3–4.1)	191 (99.0; 96.0–99.7)	0.58 (0.13–2.66)	0.48	0.44 (0.10–2.03)	0.29
Mixed	8 (2.0; 1.0–3.9)	401 (98.0; 96.1–99.0)	1.11 (0.46–2.66)	0.81	1.13 (0.44–2.93)	0.81
Asian/indigenous	0 (0.0; –)	5 (100.0; –)	–	1.00	0.00 (–)	0.99
Mental health problems						
No (reference)	18 (1.5; 1.0–2.4)	1,180 (98.5; 97.6–99.1)				
Yes	6 (3.0; 1.3–6.5)	196 (97.0; 93.5–98.7)	2.01 (0.79–5.12)	0.15	1.16 (0.43–3.10)	0.78
*Stigma M (SD; 95% CI)*	17.22 (3.4; 17.0–17.4)	17.13 (4.3; 15.3–18.9)	0.99 (0.88–1.12)	0.89	1.03 (0.91–1.16)	0.97
*Young person characteristics*						
Gender						
Male (reference)	16 (2.0; 1.23–3.24)	785 (98.0; 96.76–98.77)				
Female	8 (1.3; 0.67–2.65)	591 (98.7; 97.35–99.33)	0.66 (0.28–1.56)	0.35	0.68 (0.28–1.65)	0.39
*Age M (SD; 95% CI)*	14.46 (1.9; 13.7–15.3)	14.51 (2.0; 14.4–14.6)	0.99 (0.80–1.21)	0.90	1.05 (0.84–1.30)	0.68
Mental health condition						
None (reference)	6 (0.7; 0.31–1.54)	855 (99.3; 98.46–99.69)				
Transient	12 (3.1; 1.75–5.34)	378 (96.9; 94.66–98.25)	4.52 (1.69–12.14)	<0.01	2.78 (0.98–7.88)	0.05
Persistent	6 (4.0; 1.82–8.68)	143 (96.0; 91.32–98.18)	5.98 (1.90–18.80)	<0.01	2.49 (0.65–9.52)	0.18
*Impact of mental health condition—M (SD; 95% CI)*	1.92 (1.8; 1.1–2.7)	0.74 (1.5; 0.7–0.8)	1.32 (1.13–1.54)	<0.01	1.20 (0.98–1.48)	0.08

a Adjusted model with 1,385 participants; town of residence and data collection method (face-to-face or phone interview) were controlled.

Although not the main aim of our article, we explored whether there were any differences in service utilization among those with externalizing versus internalizing conditions. We found that having either an internalizing or externalizing diagnosis increased the odds of contact with specialized services compared with no diagnosis; however, there was no difference in contact among those with internalizing problems versus externalizing problems (Supplementary Table S4).

Among the 133 participants who used some type of service during the previous year, 63 (47.4%) reported that they wanted or needed additional support for their youth’s mental health conditions, but could not access the support (defined as sufficient care). In terms of barriers to sufficient support (Table 4), youth with persistent psychiatric diagnoses experienced more barriers to additional support (65.8%) in comparison with those with transient mental health conditions (44.8%) or no mental health condition (32.4%). Most caregivers who reported experiencing barriers to additional support described multiple barriers impeding their access to care (71.4%). Considering the four broad barrier categories, structural barriers were the most common barrier to receiving additional support (43.6%), and this was mostly due to the cost of treatment. Recognition/literacy was the second most common barrier (27.1%), and lack of knowledge about where to go or whom to trust to ask for help was the most common reason for this type of barrier. About one-quarter (25.4%) of caregivers reported lack of trust/negative experiences as an impediment to additional support, and this was mainly due to “having a previous bad experience with professionals.” Nine percent of the caregivers faced anticipated stigma experienced in this subsample (Table 4).

**Table 4.** Prevalence of barriers to receiving sufficient mental health care and support among young people who used some type of service in the past 12 months (*n* = 133^a^).

	Total service users	No mental health condition	Transient mental health condition	Persistent mental health condition
	(*n* = 133)	(*n* = 37)	(*n* = 58)	(*n* = 38)
Barrier	*n* (%; 95% CI)	*n* (%; 95% CI)	*n* (%; 95% CI)	*n* (%; 95% CI)
*At least one type*	63 (47.4; 39.0–55.9)	12 (32.4; 19.3–49.0)	26 (44.83; 32.5–57.8)	25 (65.79; 49.4–79.1)
Structural				
Cost	45 (34.1; 26.5–42.7)	8 (21.6; 11.1–37.8)	20 (35.1; 23.8–48.4)	17 (44.7; 29.8–60.7)
Distance	31 (23.3; 16.8–31.3)	6 (16.2; 7.4–31.9)	12 (20.7; 12.1–33.1)	13 (34.2; 20.9–50.6)
Waiting list	28 (21.1; 14.9–28.9)	2 (5.4; 1.3–19.4)	15 (25.8; 16.2–38.7)	11 (29.0; 16.7–45.3)
Transport/access	16 (12.0; 7.51–8.8)	3 (8.1; 2.6–22.5)	7 (12.1; 5.8–23.4)	6 (15.8; 7.2–31.1)
Other structural barrier	9 (6.8; 3.5–12.6)	2 (5.4; 1.3–9.4)	2 (3.5; 0.9–12.9)	5 (13.2; 5.6–28.1)
*Any structural*	58 (43.6; 35.4–52.2)	10 (27.0; 15.1–43.5)	24 (41.4; 29.4–54.5)	24 (63.2; 46.9–76.9)
Lack of recognition/illiteracy				
Did not know where to go	21 (15.8; 10.5–23.1)	4 (10.8; 4.08–25.70)	9 (15.5; 8.2–27.3)	8 (21.1; 10.8–36.9)
Did not know whom to trust to ask for help	17 (12.8; 8.1–19.7)	2 (5.4; 1.34–19.40)	7 (12.1; 5.8–23.4)	8 (21.1; 10.8–37.0)
Choose take care of child by her own	15 (11.3; 6.9–18.0)	5 (13.5; 5.69–28.80)	6 (10.5; 4.7–21.3)	4 (10.5; 4.0–25.1)
Child’s problem not too serious	10 (7.6; 4.1–13.6)	2 (5.6; 1.38–19.88)	5 (8.6; 3.6–19.2)	3 (7.9; 2.5–22.0)
Other recognition/literacy	4 (3.0; 1.1–7.8)	1 (2.7; 0.37–17.11)	2 (3.5; 0.9–12.9)	1 (2.6; 0.4–16.7)
Lack of trust/negative experiences				
Bad experience with professionals	22 (16.8; 11.3–24.3)	4 (10.8; 4.08–25.68)	8 (14.0; 7.1–25.8)	10 (27.0; 15.1–43.5)
Afraid that child would be taken away	12 (9.0; 5.2–15.3)	1 (2.7; 0.37–17.11)	6 (10.3; 4.7–21.3)	5 (13.2; 5.5–28.1)
Thought service would not work	10 (7.5; 4.1–13.5)	2 (5.4; 1.34–19.40)	1 (1.7; 0.2–11.4)	7 (18.4; 9.0–34.1)
People who trust say not to take to service	7 (5.3; 2.5–10.7)	3 (8.1; 2.61–22.51)	2 (3.5; 0.9–12.9)	2 (5.3; 1.3–19.0)
Other lack of trust/negative experiences	6 (4.5; 2.0–9.7)	1 (2.7; 0.37–17.11)	4 (6.0; 2.6–17.1)	1 (2.6; 0.3–16.7)
*Any lack of trust /negative experiences*	32 (24.1; 17.5–32.1)	6 (16.2; 7.42–31.87)	11 (19.0; 10.7–31.2)	15 (39.5; 25.3–55.7)
Anticipated stigma				
*Any anticipated stigma*	12 (9.0; 5.2–15.3)	2 (5.4; 1.3–19.4)	5 (8.6; 3.6–19.2)	5 (13.2; 5.5–28.1)

a Analysis with 133 out of the total 155 participants who used some type of service.

We examined associations between caregiver and youth characteristics with reporting any barrier related to receiving additional support among the subsample of service users. No caregiver or youth characteristics were associated with greater likelihood of receiving sufficient mental health care among those participants using services (Table 5).

**Table 5.** Caregiver and young person characteristics associated with barriers to sufficient mental health care among those who received at least on type of service in the past 12 months (bivariate and multivariable analyses; *n* = 133).

	Insufficient care	Sufficient care	Unadjusted analysis		Adjusted multivariable analysis^a^	
	*n* (%; 95% CI)	*n* (%; 95% CI)	OR (95% CI)	*p*-value	OR (95% CI)	*p*-value
*Caregiver characteristics*						
Education						
Primary education (reference)	25 (49.0; 35.5–62.6)	26 (51.0; 37.4–64.4)				
Secondary education	32 (48.5; 36.6–60.5)	34 (51.5; 39.5–63.4)	0.98 (0.47–2.03)	0.95	1.30 (0.56–3.00)	0.54
Higher education	5 (35.7; 15.6–62.6)	9 (64.3; 37.4–84.4)	0.58 (0.17–1.96)	0.38	0.83 (0.20–3.37)	0.79
Social class						
Middle-high (reference)	20 (40.0; 27.4–54.1)	30 (60.0; 45.9–72.6)				
Middle-low/low	43 (51.8; 41.0–62.4)	40 (48.2; 37.6–59.0)	1.61 (0.79–3.28)	0.19	1.57 (0.66–3.72)	0.31
Mental health problems						
No (reference)	44 (41.5; 32.5–51.2)	62 (58.5; 48.8–67.5)				
Yes	19 (70.4; 50.8–84.5)	8 (29.6; 15.5–49.2)	3.35 (1.35–8.33)	<0.01	2.62 (0.96–7.14)	0.06
*Young person characteristics*						
Gender						
Male (reference)	35 (43.8; 33.2–54.9)	45 (56.3; 45.2–66.8)				
Female	28 (52.8; 39.4–65.9)	25 (47.2; 34.1–60.6)	1.44 (0.72–2.89)	0.31	1.74 (0.78–3.90)	0.18
*Age M (SD; 95% CI)*	14.51 (0.3; 14.0–15.0)	14.56 (0.2; 14.1–15.0)	0.99 (0.82–1.18)	0.88	0.87 (0.70–1.10)	0.25
Mental health condition						
None (reference)	12 (32.4; 19.3–49.0)	25 (67.6; 51.0–80.7)				
Transient	26 (44.8; 32.5–57.8)	32 (55.2; 42.2–67.5)	1.69 (0.72–4.01)	0.23	1.42 (0.52–3.90)	0.49
Persistent	25 (65.8; 49.4–79.1)	13 (34.2; 20.9–50.6)	4.00 (1.53–10.47)	<0.01	2.01 (0.57–7.05)	0.28
*Impact of mental health condition—M (SD; 95% CI)*	2.6 (0.3; 1.9–3.2)	1.5 (0.2; 1.0–1.9)	1.26 (1.07–1.50)	0.01	1.24 (0.99–1.55)	0.06

a Adjusted model with 131 participants; town of residence and data collection method (face-to-face or phone interview) were controlled.

We further analyzed associations between caregiver and youth characteristics with the three most common types of barriers (structural, recognition/literacy, and lack of trust/negative experiences in services/treatments). Among those using services, three characteristics were found to be associated with respondents reporting “lack of trust/negative experiences in services/treatments” as a barrier to sufficient care: caregivers’ mental health problems (OR: 2.27; 95% CI: 0.84–6.16; *p*-value = 0.03), youth’s persistent psychopathology (OR: 3.15; 95% CI: 1.77–5.60; *p*-value < 0.01), and its greater impact on their everyday life (OR: 1.26; 95% CI: 1.06–1.49; *p*-value < 0.01). Due to small sample sizes, we consider these analyses exploratory and report them in the Supplementary Material only (Supplementary Table S5).

## Discussion

We confirmed the main hypothesis that low socioeconomic status and caregiver stigma were associated with a lower likelihood of formal or informal child mental health service use. We did not confirm that the presence of caregiver mental health problems was associated with use of the same formal/informal services.

We found that persistence of youth mental health conditions and their impact on daily life were associated with higher odds of formal care. Importantly, caregiver characteristics and beliefs were also a key influence in the care process: lower parental stigma was associated with greater formal service use, and lower SEG was associated with greater odds of informal care.

From the literature, it was expected that youth psychopathology, and caregivers’ sociodemographic characteristics, such as SEG, would represent significant barriers to care [23], particularly in LMICs such as Brazil [24].

The Brazilian health care system provides universal care for the entire population, with the mental health system being fully integrated into it. The Psychosocial Community Care Centers are treatment facilities staffed by multidisciplinary teams, and are the main source of mental health specialty care. Any Brazilian individual can seek mental health care directly from these units [25, 26]. Traditionally, most of the health funding is provided by the federal government, but in the recent years, there has been an increase in the participation of the private health system. For example, between 2015 and 2019, federal allocation decreased from 44.8 to 42.2%, whereas private health insurance (employer-subsidized plans) increased from 30.1 to 32.1%, and people themselves paying for care increased from 25.1 to 25.7% [27]. It should also be mentioned that like most LMICs and other countries in Latin America, mental health expenditure per person, as well as the number of mental health professionals, is below the recommended WHO standard [28].

It is interesting that our study found caregiver stigma to be the main barrier to access formal care for their child. Stigma associated with mental health problems represents a substantial burden to individuals, families, and society, being described as one of the most relevant barriers to care in different countries and cultures [29]. Parental attitudes and beliefs are particularly relevant due to their role as a key gatekeeper to their child’s treatment access. Therefore, caregivers’ perceptions may influence concerns around labeling related to their child’s condition, and their perceptions of treatment options, impacting the help-seeking trajectory [7, 9].

At the same time, there are several effective interventions which can reduce help-seeking stigma, particularly encouraging are those involving different forms of social contact [30]. Despite this, most studies were carried out in high-income countries and with adults [31]. Moreover, there is a paucity of research looking at the role of parental stigma on youth mental health service use. One study from the United Kingdom [7] found that the odds of service use were greater among caregivers with less intended stigmatizing behaviors. Another study in the United States identified higher social distance among parents with lower mental health problem recognition [32]. More recently, a cohort of young people from the United Kingdom identified that their persistent psychopathology, family socioeconomic disadvantage, and low caregiver intended stigma-related behavior were associated with increased likelihood of youth service use, whereas young people older age and socioeconomic disadvantage were associated with increased costs [33].

Although the number of participants reporting informal service use was small in our sample, it represents a potentially important source of support and an area where little research has been conducted. We found this result surprisingly rare, knowing that families usually experience a wide variety of pathways to care which often includes informal care in regions and countries with fewer services [4]. Among those who used informal care, the vast majority with a mental health condition (almost 90%) also reported formal service use. This suggests that informal care did not prevent access to formal care [34], although we do not know if informal care led to formal care [35].

The type of informal care reported in our sample is similar to that reported in other studies, with religious services being the most frequently stated by Latino samples [36]. Moreover, our rates of contact with religious providers for mental health care and complementary contacts with formal services were similar to those found among adult participants of the World Mental Health Survey [37].

We also found that low SEG was the only significant predictor of greater informal care utilization. Caregivers from low SEGs might have easier access to and comfort with using informal care compared to formal mental health services since religious and other informal sources are usually free of charge [37, 38] and quite available in Brazil, a strongly religious country. These types of services may also be seen as less stigmatizing than more traditional formal mental health services. Moreover, in addition to mental health problems, these individuals may be more likely to experience social problems and complex circumstances, which may limit accessibility to formal care, including out of pocket payments, lack of health insurance, and difficulties in getting to the service [9, 39].

The low informal care utilization in the current study may also have other explanations. It is possible that caregivers use informal care, but do not identify these sources of care as providing mental health support, or they may not consider these types of informal providers as capable of dealing with mental health problems and thus prefer formal care. Additionally, our definition of informal care was focused on nontraditional providers which could deliver some services and support, but did not include family and friends which can also be an informal source of help [34, 35]. Although our results are novel, the numbers are very low (only 24 youth used informal care); thus, further studies are necessary to understand factors related to informal care.

No other youth characteristics (age or gender) impacted informal or formal service utilization in our sample. Previous studies from Brazil and other countries suggest that service use may vary according to gender, age, and type of condition. For example, boys with externalizing symptoms access services more often during childhood, whereas girls with internalizing problems receive more help during adolescence [40–42]. There is no consensus in the field about these relationships as they also depend on sociocultural context; however, we also did not have the power to explore the interactions between these different variables and how they may change as the child ages and we recognize this is a limitation of our study.

As described above, at least in high-income countries, there is good evidence about barriers related to nonattendance [43] as well as treatment dropout [44], but less is known about barriers to care among those using some type of service [44, 45]. The next step of our analysis was to understand the main barriers among the subsample of youth already using services and needing additional care. While just under one-third of those with persistent and impactful problems received any care, the majority of those in care reported needing more support and experienced barriers to sufficient care. Thus, stigma was important for getting people into care, while structural barriers were the most common obstacle to achieve sufficient care.

Among those using services, the majority did not feel that the care was adequate and structural barriers (mainly high cost, distance, and waiting lists) were the most significant impediments to receiving sufficient care. Hence, stigma was less important, suggesting that after families were able to access treatment, stigma decreased its power as a barrier. Another hypothesis is that people already consulting services had low stigma; therefore, the other types of barriers were more relevant for them.

Structural barriers are well known as being one of the main problems in respect of accessing mental health care. Increased financial investments, better coordination of the existing resources, the training of health providers, and empowering professionals and parents are some of the strategies recommended to improve the quality of youth mental health provision [8, 9, 26, 46].

Our study did not identify any barriers to sufficient mental health care related to caregiver or the characteristics of the young people. Our exploratory analysis, however, identified weak evidence of two potential barriers which may require further investigation: the presence of caregiver mental health problems, and mental health conditions that have a significant effect on the everyday lives of the youths. Families with a child with more severe dysfunction and the presence of parent psychopathology might result in more complex needs due to a higher number of problems [6, 44, 45]. Moreover, potential burden associated with the child’s problems may be amplified in the presence of a caregiver’s own mental health problems [6]. Given the shortage of mental health specialists everywhere [47], including in Brazil [8, 26], access to sufficient care is more challenging for complex cases who need more intense and specialized help [29, 44]. Further studies with larger sample sizes are required to verify these hypotheses.

The current study has some limitations. There were some differences between the original sample and the final one at the follow-up due to attrition rates. Even though not ideal, this does not seem to be a major issue because our study is not a prevalence or incidence study, where representative samples would be mandatory. Although we used a validated instrument which was adapted to the Brazilian context, data related to service use were limited to caregiver reports. However, the concordance between parent report and records of service use on the parental version of SACA is strong [48]. There may also be underreporting of informal care, given the interviewers could be perceived as scientists or mental health professionals. Parental stigma was assessed after service contact; therefore, it is difficult to establish the direction of the effect of stigma on service use. The frequency of service utilization was small; therefore, results should be taken with caution and additional studies are needed to replicate these findings, especially from informal care and related to barriers to sufficient care. Finally, the young person’s mental health condition at baseline was reported exclusively by their caregivers; the inclusion of the young person’s own perspective in respect of their mental health would have increased the accuracy of our data.

Even with these limitations, there are few studies with detailed data on barriers to accessing youth mental health services, using validated measures of service use covering a wide range of types of formal and informal care among a large community sample. It is noteworthy that this is one of the few studies looking at youth mental health service use in an LMIC.

In conclusion, our results reinforce the relevance of caregivers on the help-seeking process to access services for youth with mental health problems. More specifically, the role of parental stigma was a key barrier to formal care and low socioeconomic position to informal care. Thus, generating data about effective interventions to reduce stigma and promote access to coordinated care are important priorities worldwide, and particularly among vulnerable populations.

## Data Availability

Data were provided by the Brazilian High-Risk Cohort Study and are available upon request in the Open Science Framework public repository (https://osf.io/ktz5h/).

## Supplementary material

For supplementary material accompanying this paper visit https://doi.org/10.1192/j.eurpsy.2022.24. click here to view supplementary material
