# Supplementary material for: The influence of caregiver attitudes and socioeconomic group on formal and informal mental health service use among youth
Source: Eur Psychiatry. 2022 Jun 10;65(1):e34. doi: 10.1192/j.eurpsy.2022.24 (PMC9251818; doi:10.1192/j.eurpsy.2022.24)
Supplement: Supplementary file 1 [file S0924933822000244sup001.docx]

BHRC

Neuroimaging

N=680

13 interviews with incomplete data

Total telephone

interviews

N=995

Telephone

Interviews N=970

BHRC

First Follow-up participants

N=2,010

Complete

Face-to-face Interviews N=418

Complete

telephone interviews

N=982

Telephone

Interviews N=25

Face-to-face Interviews N=418

BHRC

No-neuroimaging

N=1,201

BHRC First follow-up

participants invited in the health-related service use study

N=1,881

Total mental-health related service use sample =1,400

BHRC

Baseline participants

N=2,511

**Supplementary Figure 1.** Flow chart of Brazilian High-Risk Cohort participants included in the mental health-related service use study

**Appendix 1.** Comparison of characteristics among baseline BHRC participants and health-related service use participants.

|  | **Baseline BHRC**  **participants**  **N 1,111 (%)** | **Health-related service use participants**  **N 1,400 (%)** | **OR (95% CI)** | **p-value** |
| --- | --- | --- | --- | --- |
|  |  |  |  |  |
| ***CAREGIVER CHARACTERISTICS*** | | | | |
| **Education** | | | | |
| Primary education (reference) | 489 (48.7)* | 515 (51.2)****** |  |  |
| Secondary education | 529 (41.7) | 739 (58.3) | 0.75 (0.64-0.89) | 0.001 |
| Higher education | 82 (38.9) | 129 (61.1) | 0.67 (0.49-0.91) | 0.01 |
| ***Social Class*** | | | | |
| Middle-High (reference) | 413 (41.4)^&^ | 585 (58.6) |  |  |
| Middle-Low/Low | 698 (46.1) | 815 (53.9) | 1.21 (0.62-0.80) | 0.02 |
| ***Ethnicity***** | | | | |
| White (reference) | 662 (45.6)^#^ | 790 (54.4)^##^ |  |  |
| Black | 157 (44.9) | 193 (55.1) | 0.97 (0.77-1.23) | 0.80 |
| Mixed | 275 (40.2) | 409 (59.8) | 0.80 (0.67-0.97) | 0.02 |
| Asian/indigenous | 14 (73.7) | 5 (26.3 | 3.34 (1.20-9.33) | 0.02 |
| ***YOUNG PERSON CHARACTERISTICS*** | | | | |
| ***Gender*** | | | | |
| Male (reference) | 574 (41.8) | 801 (58.3) |  |  |
| Female | 537 (47.3) | 599 (52.7) | 1.25 (1.07-1.47) | 0.01 |
| ***Age M (SE*)** | 10.29 (0.05) | 10.29 (0.06) | 1.05 (1.00-1.09) | 0.04 |
| ***Mental health condition*** | | | | |
| No (reference) | 821 (44.2) | 1,038 (55.8) |  |  |
| Yes | 290 (44.5) | 362 (55.2) | 1.01 (0.85-1.21) | 0.89 |
| ***Impact of mental health condition***  ***– M (SE*)** | 0.94 (0.05) | 0.91 (0.04) | 1.01 (0.96-1.07) | 0.62 |

*11 missing data **7 missing data; ^&^ 8 missing data; ^#^ 2 missing data; ^##^ 3 missing data

**Appendix 2.** Services classification by formal and informal, and barriers to sufficient mental health care

| **FORMAL** | | | | |
| --- | --- | --- | --- | --- |
| ***Health*** | | | | |
| **Inpatient** | psychiatric hospital | psychiatric bed in general hospital | overnight stay at alcohol/drug clinic | therapeutic residence |
| **Emergency** | emergency department |  |  |  |
| **Specialty/specialist outpatient mental health care** | outpatient mental health clinic | outpatient alcool/drug clinic | mental health specialist (psychiatrist, psychologist, mental health social worker, family counselor) |  |
|  |  |  |  |  |
| **General outpatient care** | primary care clinic | non specialist (paediatrician, general practitioner or family doctor) |  |  |
| ***Education*** | | | | |
| **Therapy at school** | counselling | therapy |  |  |
| **School assistance** | classroom assistant | classroom support |  |  |
| **Special classroom** |  |  |  |  |
| **Special school** |  |  |  |  |
| **INFORMAL** | | | | |
| **Religious** | priest | pastor | healer | other religious leader |
| **Self-help** | suicide hotline | peer support groups |  |  |
| **Alternative** | acupuncture | chiropractor |  |  |
| **BARRIERS TO SUFFICIENT CARE*** | | | | |
| **Structural barriers** | Distance from the service | Cost | Availability of treatment |  |
| **Recognition & literacy** | Did not know where to find a service/treatment | Did not know whom to trust | Preference to treat the problem themselves | Considering the problem to be not too serious |
| **Lack of trust or Negative experiences on services/treatment** |  |  |  |  |
| **Anticipated stigma** | Worried about what family and friends would say |  |  |  |

*All “other” barriers were reclassified into one of these four categories

**Appendix 3.** Sample description overall and by the trajectory of mental health condition (n=1,400)

| **Sociodemographic characteristics** | **Full sample** | **No mental health condition** | **Transient MH problem** | **Persistent MH problem** | **p-value** |
| --- | --- | --- | --- | --- | --- |
|  | **(n=1,400)** | **(n=858)** | **(n=387)** | **(n=148)** |  |
|  | **n (%)** | **n (%)** | **n (%)** | **n (%)** |  |
| **CAREGIVER CHARACTERISTICS** | | | | | |
| ***Education**** | | | | | |
| Primary education | 620 (44.5) | 387 (45.1) | 174 (45.0) | 59 (39.9) | 0.79 |
| Secondary education | 626 (44.9) | 384 (44.8) | 171 (44.2) | 71 (48.0) |  |
| Higher education | 147 (10.6) | 87 (10.1) | 42 (10.9) | 18 (12.2) |  |
|  |  |  |  |  |  |
| ***Social class*** | | | | | |
| Middle-High | 554 (39.6) | 359 (41.7) | 134 (34.4) | 61 (40.9) | 0.05 |
| Middle-Low/Low | 846 (60.4) | 502 (58.3) | 256 (65.6) | 88 (59.1) |  |
|  |  |  |  |  |  |
| ***Ethnicity***** | | | | | |
| White | 790 (56.6) | 484 (56.2) | 222 (57.1) | 84 (57.1) | 0.47 |
| Black | 193 (13.8) | 111 (12.9) | 61 (15.7) | 21 (14.3) |  |
| Mixed | 409 (29.3) | 261 (30.3) | 106 (27.3) | 42 (28.6) |  |
| Asian/indigenous | 5 (0.5) | 5 (0.6) | 0 (0.0) | 0 (0.0) |  |
|  |  |  |  |  |  |
| **YOUNG PERSON CHARACTERISTICS** | | | | | |
| ***Gender*** | | | | | |
| Male | 801 (57.2) | 503 (58.4) | 215 (53.1) | 83 (55.7) | 0.51 |
| Female | 599 (42.8) | 358 (41.6) | 175 (44.9) | 66 (44.3) |  |
|  |  |  |  |  |  |
| ***Age*** ***(SD****)* | 14.5 (1.9) | 14.5 (2.0) | 14.5 (1.9) | 14.7 (2.0) | 0.57 |

* 7 missing data; **3 mssing data

**Appendix 4**. Any type and specialty/specialist outpatient mental health service use in the past 12 months among Brazilian young people by broad diagnostics groups of mental health conditions at first follow-up (n=1,396)^a^

|  | **Any type of service** | **Unadjusted Analysis** | | **Adjusted Multivariable Analysis*** | |
| --- | --- | --- | --- | --- | --- |
|  | **n (%; 95% CI)** | **OR (95% CI)** | **p-value** | **OR (95% CI)** | **p-value** |
| None (reference) (n=1,074) | 78 (7.3; 5.9 - 9.0) | ----- |  | ------ |  |
| Internalising^#^ (n=205) | 35 (17.1; 13.0 – 22.9) | 2.63 (1.71- 4.04) | <0.01 | 1.61 (0.98- 2.62) | 0.06 |
| Externalising^##^ (n=72) | 25 (34.7; 24.6 - 46.4) | 6.79 (3.97-11.62) | <0.01 | 2.66 (1.41-5.02) | <0.01 |
|  |  |  |  |  |  |
| Comorbid (n=45)^###^ | 15 (33.3; 21.1 - 48.4) | 6.39 (3.30-12.37) | <0.01 | 1.94 (0.86-4.38) | 0.11 |
|  | **Specialty/**  **outpatient service** | **Unadjusted Analysis** | | **Adjusted Multivariable Analysis*** | |
|  | **n (%; 95% CI)** | **OR (95% CI)** | **p-value** | **OR (95% CI)** | **p-value** |
| None (reference) (n=1,074) | 53 (4.9; 3.8-6.4) | ------ |  | ------ |  |
| Internalising^#^ (n=205) | 31 (15.1; 10.8-20.7) | 3.43 (2.14-5.50) | <0.01 | 2.19 (1.29-3.73) | <0.01 |
| Externalising^##^ (n=72) | 19 (26.4; 17.4-37.8) | 6.91 (3.82-12.49) | <0.01 | 2.70 (1.34-5.45) | <0.01 |
|  |  |  |  |  |  |
| Comorbid (n=45)^###^ | 12 (26.7; 15.7-41.5) | 7.01 (3.42-14.34) | <0.01 | 2.07 (0.85-5.04) | 0.11 |

^a^Participants with other thought disorders (n=3) and psychosis (n=1) were excluded from these classifications due to divergent literature as to which broad group they might belong.

^#^Internalising mental health condition includes distress-related diagnosis: depression, generalized anxiety disorder, obsessive–compulsive disorder, tic, eating disorder, and fear-related disorders: panic, agoraphobia, social anxiety, specific phobia, and separation anxiety)

^##^Externalising mental health condition includes conduct disorder, oppositional defiant disorder, and attention deficit/hyperactivity disorder [ADHD]).

^###^Comorbidity was generated for individuals with internalising and externalising diagnoses.

*Adjusted model with 1,381 participants; caregiver’s education, social class, ethnicity, and mental health problem; youth’s gender and age; town of residence and data collection method (face to face or phone interview) were controlled.

**Appendix 5**. Caregiver and child/adolescent characteristics associated with specific types of further barriers to sufficient care among those who received at least on type of service in the past 12 months (logistic regression) (n=131)

|  | **STRUCTURAL** | | | | **RECOGNITION & LITERACY** | | | | **LACK OF TRUST & NEGATIVE EXPERIENCES IN SERVICES/TREATMENTS** | | | |
| --- | --- | --- | --- | --- | --- | --- | --- | --- | --- | --- | --- | --- |
|  | **(n=** 58**)** | | | | **(n= 36)** | | | | **(n= 32)** | | | |
|  | Unadjusted OR **(95% CI)** | p-value | Adjusted OR **(95% CI)** | p-value | Unadjusted OR **(95% CI)** | p-value | Adjusted OR **(95% CI)** | p-value | Unadjusted OR **(95% CI)** | p-value | Adjusted OR **(95% CI)** | p-value |
| **CAREGIVER CHARACTERISTICS** | | | | | | | | | | | | |
| ***Education*** | | | | | | | | | | | |  |
| Primary education (reference) |  |  |  |  |  |  |  |  |  |  |  |  |
| Secondary education | 0.83 (0.40-1.73) | 0.62 | 1.12 (0.48-2.63) | 0.79 | 0.99 (0.44-2.25) | 0.98 | 0.92 (0.38-2.25) | 0.86 | 0.65 (0.28-1.50) | 0.31 | 0.62 (0.24-1.57) | 0.31 |
| Higher education | 0.89 (0.18- 2.13) | 0.45 | 1.1 (0.270-4.56) | 0.89 | 0.72 (0.18-2.97) | 0.65 | 0.86 (0.18-4.19) | 0.85 | 0.66 (0.16-2.69) | 0.56 | 0.76 (0.15-3.88) | 0.75 |
|  |  |  |  |  |  |  |  |  |  |  |  |  |
| ***Mental health problem*** | | | | | | | | | | | | |
| No (reference) |  |  |  |  |  |  |  |  |  |  |  |  |
| Yes | 3.3 (1.35-8.05) | <0.01 | 2.55 (0.94-6.86) | 0.07 | 2.23 (0.92-5.42) | 0.08 | 1.75 (0.66-4.67) | 0.26 | 2.78 (1.13-6.87) | **0.03** | 2.27 (0.84-6.16) | 0.11 |
|  |  |  |  |  |  |  |  |  |  |  |  |  |
| ***Social class*** | | | | | | | | | | | | |
| Middle-High (reference) |  |  |  |  |  |  |  |  |  |  |  |  |
| Middle-Low/Low | 2.19 (1.05-4.53) | 0.04 | 2.36 (0.95-5.83) | 0.06 | 1.29 (0.58-2.88) | 0.62 | 1.31 (0.51-3.37) | 0.58 | 1.2 (0.52-2.76) | 0.43 | 1.06 (0.39-2.84) | 0.91 |
| **CHILD/ADOLESCENT CHARACTERISTICS** | | | | | | | | | | | | |
| ***Gender*** | | | | | | | | | | | | |
| Male (reference) |  |  |  |  |  |  |  |  |  |  |  |  |
| Female | 1.44 (0.72-2.91) | 0.3 | 1.67 (0.73-3.83) | 0.22 | 1.3 (0.60-2.82) | 0.51 | 1.19 (0.50-2.86) | 0.4 | 1.24 (0.55-2.77) | 0.61 | 1.1 (0.44-2.77) | 0.84 |
|  |  |  |  |  |  |  |  |  |  |  |  |  |
| ***Age - M (SD****)* | 1.02 (0.85-1.22) | 0.85 | 0.9 (0.71-1.13) | 0.35 | 1.11 (0.91-1.36) | 0.32 | 1.08 (0.84-1.38) | 0.55 | 1.02 (0.82-1.25) | 0.92 | 1.03 (0.80-1.33) | 0.81 |
| ***Psychiatric diagnosis*** | | | | | | | | | | | | |
| None (reference) |  |  |  |  |  |  |  |  |  |  |  |  |
| Transient | 1.91 (0.78-4.66) | 0.16 | 1.36 (9.48-3.87) | 0.56 | 1.36 (0.49-3.78) | 0.55 | 1.42 (0.47-4.34) | 0.54 | 1.21 (0.41-3.61) | 0.73 | 0.88 (0.26-3.02) | 0.85 |
| Persistent | 4.63 (1.74-12.34) | <0.01 | 2.22 (0.62-8.02) | 0.22 | 2.8 (1.98-7.98) | 0.06 | 3.23 (0.82-12.71) | 0.09 | 3.37 (1.13-10.02) | **0.03** | 1.65 (0.40-6.73) | 0.49 |
|  |  |  |  |  |  |  |  |  |  |  |  |  |
| ***Impact of mental health problems – M (SD)*** | 1.27 (1.07-1.50) | <0.01 | 1.24 (1.00-1.55) | 0.05 | 1.01 (0.85-1.20) | 0.89 | 0.9 (0.72; 1.12) | 0.34 | 1.26 (1.06-1.49) | **0.01** | 1.18 (0.95-1.48) | 0.14 |

*Adjusted model with 131 participants; town of residence and data collection method (face to face or phone interview) were controlled.
